# The role of FACT in managing chromatin: disruption, assembly, or repair?

**DOI:** 10.1093/nar/gkaa912

**Published:** 2020-10-26

**Authors:** Tim Formosa, Fred Winston

**Affiliations:** Department of Biochemistry, University of Utah School of Medicine, Salt Lake City, UT 84112, USA; Department of Genetics, Harvard Medical School, Boston, MA 02115, USA

## Abstract

FACT (FAcilitates Chromatin Transcription) has long been considered to be a transcription elongation factor whose ability to destabilize nucleosomes promotes RNAPII progression on chromatin templates. However, this is just one function of this histone chaperone, as FACT also functions in DNA replication. While broadly conserved among eukaryotes and essential for viability in many organisms, dependence on FACT varies widely, with some differentiated cells proliferating normally in its absence. It is therefore unclear what the core functions of FACT are, whether they differ in different circumstances, and what makes FACT essential in some situations but not others. Here, we review recent advances and propose a unifying model for FACT activity. By analogy to DNA repair, we propose that the ability of FACT to both destabilize and assemble nucleosomes allows it to monitor and restore nucleosome integrity as part of a system of chromatin repair, in which disruptions in the packaging of DNA are sensed and returned to their normal state. The requirement for FACT then depends on the level of chromatin disruption occurring in the cell, and the cell's ability to tolerate packaging defects. The role of FACT in transcription would then be just one facet of a broader system for maintaining chromatin integrity.

## INTRODUCTION

FACT (FAcilitates Chromatin Transcription) is a broadly conserved histone chaperone that was named for its ability to promote elongation by RNA Pol II through nucleosomes *in vitro* (1). However, FACT appears to function in at least two fundamental processes, as it associates with transcription machinery but it also directly contacts DNA replication complex components, including the replicative helicase MCM2–7 and DNA polymerase α (2–7). FACT function is important for repressing transcription initiation from some promoters, for preserving histone modification patterns during transcription (8–12) and for establishing functional centromeres (13–16), suggesting that it establishes or stabilizes chromatin. However, FACT also enhances nucleosome eviction during promoter activation, and can weaken nucleosomal barriers to RNA and DNA polymerase progression, suggesting that it removes or destabilizes chromatin (17–22).

Genetic analysis reveals that FACT is essential in a broad range of eukaryotic organisms (2,23–24). However, some differentiated cell types proliferate normally without FACT, indicating that neither transcription nor DNA replication are fully dependent on it under all circumstances (24,25). Notably, mammalian cells lacking FACT appear to be unable to make transitions between differentiation states (24–27), suggesting a role in reprogramming chromatin. Cancer cells appear to require higher FACT activity than normal cells, so the importance of FACT activity may vary with circumstances (28–32). FACT is therefore implicated in multiple processes that involve establishing or managing the properties of chromatin, but the importance of its different roles is not constant among species or even among cell types within a single species during different stages of normal or aberrant differentiation. It remains unknown why it is sometimes essential, which of its roles is required in these cases, or even whether it is the same role that is most important in each cell type.

**Figure 1.**
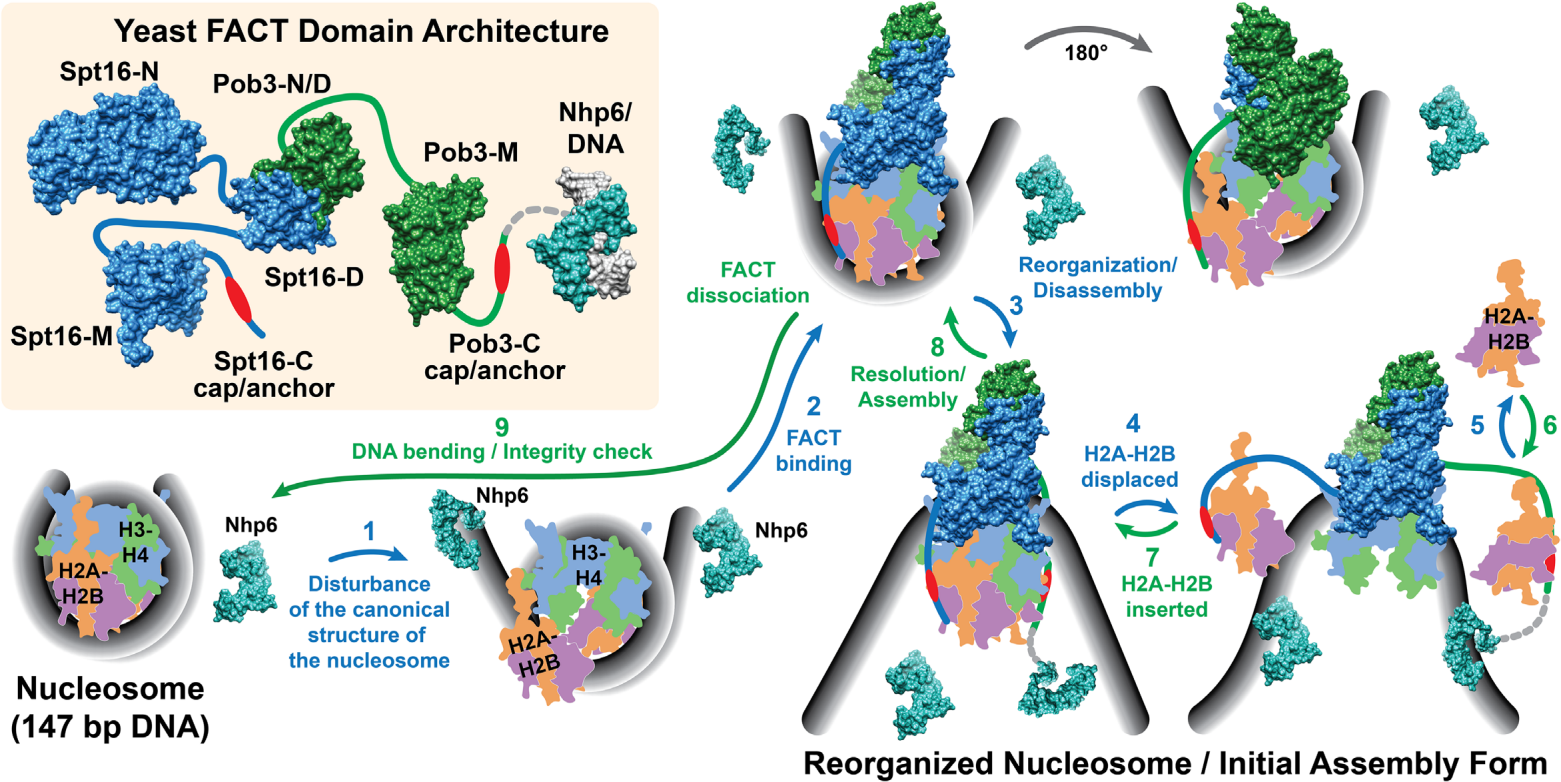
Potential steps in nucleosome destabilization and assembly/repair assisted by FACT. Surface representations of yeast Spt16, Pob3 and Nhp6:DNA are shown in the upper left panel. A dotted gray line indicates the connection between SSRP1-M and the HMGB1 domain in human SSRP1 that is not found in yeast. The N-terminal domains of Spt16 and Pob3 (N), the Spt16:Pob3 dimerization domains (D), middle domains (M) and C-terminal domains (C) are shown, with the ‘cap/anchor’ region within each of the acidic tails of Spt16-C and Pob3-C that bind H2B indicated by red ovals (54). Thick lines indicate the connections made by flexible, inherently disordered linkers that were not visible in the individual domain structures. The remaining panels illustrate potential steps in the reversible disassembly (blue arrows) and assembly (green arrows) of a nucleosome. (1) A canonical nucleosome (bottom left) is partially disrupted by transcription, replication, repair or some other process (including normal ‘breathing’ of the DNA:histone contacts captured by Nhp6 or other HMGB1 family members). This leads to dissociation of the DNA:(H2A-H2B) contact at the entry/exit points, and potential dislocation of the H2A-H2B dimer association with H3-H4. (2) Partial unwrapping of the DNA or partial displacement of H2A-H2B exposes binding sites for the cap/anchor modules of the acidic C-terminal domains of Spt16 and Pob3 on H2B (top, right, based on the cryo-EM structure of human FACT bound to a partial nucleosome, which did not define the Spt16-N location so it is omitted here; (45) and see Figure 2). (3) Further unwrapping of the DNA can either lead to binding of FACT to an intact octameric form or (4) promote release of an H2A-H2B dimer, exposing the binding sites for the Spt16-M and Pob3-M domains on H3-H4 in either case. The resulting reorganized nucleosome (bottom, right) retains the original components, now dissociated from one another but tethered together by FACT, but can also lead to full dissociation of an H2A-H2B dimer (5) and replacement from the pool of dimers (6). The process is reversible (7, 8), so a reorganized nucleosome can be resolved to a canonical form without disturbing the original composition or modification state of the nucleosome, or other histone chaperones could populate a FACT:DNA complex with histones to initiate *de novo* assembly from the same point. We propose that the final dissociation of FACT (9) occurs only if the nucleosome is properly assembled, providing a monitor for nucleosome integrity. Nhp6 is shown in potential locations to promote bending of the DNA during both disassembly and assembly, with the HMGB1 domain location illustrated in some cases to show its potential position in SSRP1 (dotted gray lines). Figure panels are based on PDB IDs: 1J5N (Nhp6-DNA, 53), 4KHB (Pob3 N/D-Spt16 D, 59), 2GCL (Pob3-M, 127), 3BIQ (Spt16-N, 42), 4IOY (Spt16-M, 141), 4Z2M (Spt16-M:(H3-H4)_2_, 48), 4WNN (Spt16-C:H2A-H2B, 54) and 6UPK, 6UPL (human FACT with partial nucleosomes, 45) and rendered in Chimera (142). The nucleosome is a cartoon representation derived from 1ID3 (143).

Initial models for FACT function focused on its ability to convert canonical nucleosomes into a less stable, but stoichiometrically complete, form *in vitro*. Because the nucleosome took on distinct new properties when bound by FACT but had the same components as a canonical nucleosome, this activity was named ‘reorganization’ (2,33, Figure 1). This ability to disrupt stable nucleosomes in the absence of adenosine triphosphate hydrolysis supported models in which FACT enhances access to DNA, such as during progression of RNA or DNA polymerases or activation of repressed promoters. However, reorganization of nucleosomes by FACT is reversible and can therefore also result in the assembly of canonical nucleosomes from mixtures of histones and DNA (17,34). Thus, FACT might participate in chromatin deposition *de novo* during DNA replication, perhaps by parsing histones deposited in a less ordered state by other chaperones into sets with the correct stoichiometry, by directly assembling nucleosomes, or by monitoring the quality of the products. It could have a similar range of functions during transcription, helping to maintain or restore the integrity of nucleosomes. FACT might therefore function by disrupting chromatin in some settings but by establishing or repairing it in others, with different types of cells having greater need for one of these functions in different situations.

Several recent reviews have focused on structural features of FACT and its roles in development and carcinogenesis (23–24,29,35). Here, we review results that support a broader role for FACT in balancing the construction and deconstruction of chromatin, and how this affects the regulation of transcription as well as other chromatin-centered processes including DNA replication and repair. We also discuss potential explanations for the differential dependence of eukaryotic cells on these functions of FACT and propose that this variability may result from different tolerance for chromatin disruption and, by analogy with DNA repair, different degrees of dependence on ‘chromatin repair’ mediated by FACT.

## STRUCTURE AND FUNCTION OF FACT

**Figure 2.**
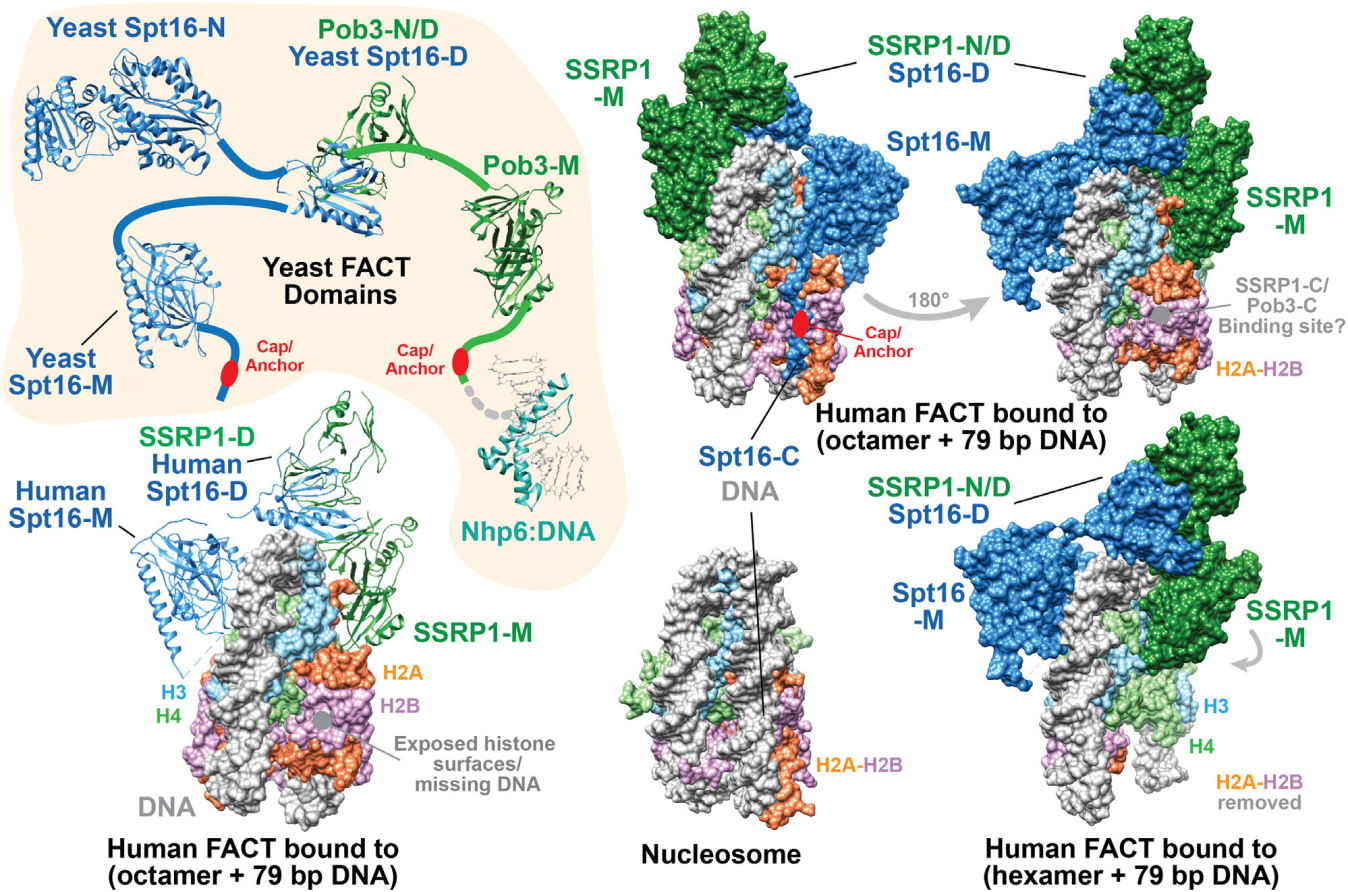
Structures of the domains of FACT alone and bound to a partial nucleosome. Individual domains of yeast FACT are shown in the upper left panel as in Figure 1, except as ribbon diagrams. The bottom left panel shows the structure of human FACT bound to a histone octamer wrapped by a 79 bp DNA fragment (45), with ribbon representations of the FACT domains whose positions were visible in the EM reconstructions; the yeast domains in the upper left are oriented to mimic the positions of the human FACT domains in the bound complex to highlight the similarity. The right panels show the hFACT:(partial nucleosome) complex in surface view. The top left view shows an orientation that highlights the placement of the Spt16-C domain along the track that would normally be occupied by DNA, with a full nucleosome below it in the same orientation for comparison. The top right panel, shows the flipped view of the complex with an octameric core, indicating the potential site that could be occupied by the similar C-terminal domain of Pob3/SSRP1. The bottom right panel shows the same orientation of the complex lacking an H2A-H2B dimer, illustrating the rotation of the SSRP1-M domain as it contacts an H2A-H2B surface in the octamer, and with H3-H4 histone surfaces exposed by removal of the H2A-H2B dimer in the hexasome. Panels are based on the structures indicated in Figure 1.

FACT is a heterodimer of two proteins, Spt16 and SSRP1 in metazoans and Spt16 and Pob3 in yeast and fungi (2,24,35–37; Figures 1 and 2). The Spt16 subunit has a similar domain composition in all species, but metazoan SSRP1 includes an HMGB1-class DNA-binding domain at its C-terminus that is not found in Pob3 (Figure 2). Instead, the HMGB1-domain protein Nhp6 supports yeast FACT activity *in vitro* and *in vivo* as a part of the yeast FACT–nucleosome complex (38–40).

The structures of individual domains of FACT have been determined, and these domains appear to be connected to one another by unstructured, flexible linkers (Figures 1 and 2). The N-terminal domain of Spt16 is highly conserved, and has homology to bacterial aminopeptidases (41,42). Consensus active site residues are missing and no peptidase activity has been detected, suggesting that this domain may function by binding to the amino-terminal regions of proteins, possibly those of the histones. However, the NTD can be deleted in yeast cells without severe effects (43), so its role in FACT function is both non-essential and poorly understood. The other structured domains of FACT are all based on a similar pleckstrin-homology architectural motif that can form binding sites for a broad range of substrate chemistries (44). FACT therefore contains multiple domains that can bind other factors at least partially independently from one another, suggesting that FACT could bind multiple components of nucleosomes and tether them together to prevent their dispersal or hold them in proximity to one another to promote assembly.

Support for this idea comes from a recent cryo-EM structure that provides a first look at how the individual domains of FACT cooperate with one another during nucleosome binding (35,45, Figure 2). Two related complexes were observed, each with an overall ‘unicycle’ architecture, with the Spt16:SSRP1 dimerization domain contacting the edge of the nucleosome near the H3:H3 interface as the seat of the unicycle, and the middle domains of both Spt16 and SSRP1 extending like the forks of the unicycle to contact each face of the nucleosome as the pedals. The dimerization domain therefore connects the Spt16 and SSRP1 subunits to one another as expected, but it also makes direct contacts with the nucleosome, and appears to organize or perhaps even coordinate the actions of the middle domains.

One of the complexes that was detected contained a full histone octamer while the other lacked one H2A-H2B dimer, suggesting that FACT promotes interconversion between octameric and hexameric histone cores (35,45). This helps to resolve differences among published reports in which some studies suggested that displacement of an H2A-H2B dimer is an obligate part of FACT function and could therefore be blocked by cross-linking the histones together, while others found that FACT can function while retaining all components of the octamer and is minimally affected by cross-linking (17,21,34,46–48). The two structures show that either interpretation is plausible depending on which stage of reorganization is achieved during a given process. A conformational change in which the middle domain of SSRP1 is repositioned between the two forms (Figure 2) indicates that FACT binding is dynamic, altering its contacts as the nucleosome undergoes structural shifts and binding sites become available or are obscured. This suggests an interactive choreography, with FACT promoting some structural maneuvers of histone domains and DNA while blocking others that might be inconsistent with the canonical assembly pathway. Single-particle FRET experiments also support central aspects of this model, showing that FACT reversibly uncoils DNA from nucleosomes and that FACT mutations and histone mutations that suppress their phenotypes *in vivo* also alter the distribution between canonical and reorganized forms *in vitro* (49–51). The kinetics of dissociation of complexes is therefore affected by both the functional integrity of FACT domains and the ability of histones to adopt the canonical nucleosomal structure.

The role of the HMGB1 domain in FACT function and the reasons for having two distinct architectures with separate Pob3 plus Nhp6 in some cases and the fused SSRP1 configuration in others remain under investigation. Nhp6 is typical of HMGB1 family members in that it binds in the minor groove of DNA in a largely sequence-independent manner, with the DNA axis bent over 60° in the complex (52,53; see Figures 1 and 2). This suggests that the role of the HMGB1 domain might be to stabilize a bent or kinked form of DNA, perhaps promoting breathing of the DNA at the entry/exit points to expose histone surfaces. Consistent with this, the cryo-EM structure described above was obtained with subnucleosomal, 79 bp DNA fragments that leave the entry/exit site histone contact sites exposed (34,45; Figure 2). The acidic C-terminal domain of Spt16 follows the track of the missing DNA (45), positioning the ‘cap and anchor’ region to make the previously described contact with H2B (54). The C-terminal tails of SSRP1 and Pob3 also have this feature, and could presumably compete with the DNA at the symmetrical site, although this was not observed in the cryo-EM structure and therefore does not seem to be as stable or uniquely positioned as the Spt16-C domain (Figure 2). A DNA-binding/bending module therefore seems to be an important component of FACT activity, possibly to enhance the exposure of the binding sites for the C-terminal tails of SSRP1/Pob3 and Spt16. However, another consideration is that the DNA in mature nucleosomes is strongly curved, so nucleosome assembly must include a way to overcome the inherent stiffness of the DNA to establish DNA:histone contacts. HMGB1 proteins have been called ‘DNA chaperones’ because they stabilize bent forms of DNA and therefore increase the rate at which sequences separated by short distances in the DNA interact with one another. This could enhance the efficiency of nucleosome assembly by producing the curvature in the DNA needed to wrap around the histone octamers (55–57). The HMGB1 domain could therefore promote exposure of FACT binding sites as an early step in reorganization, and also enhance bending of the DNA to contribute to a late step in nucleosome assembly (56; Figure 1). Nhp6 may also act directly on FACT by altering the conformation of the other domains relative to one another by binding to the C-terminal tails (58).

Yeast Spt16:Pob3, when supplemented with Nhp6, is able to form stable complexes with nucleosomes that display decreased electrophoretic mobility, increased accessibility of the DNA to many factors including restriction endonucleases, and decreased H2A-H2B content resulting in a mixture of hexameric and octameric forms (2). The DNA is unwound from the histone core during this reorganization reaction, as detected by loss of FRET signal in single-particle measurements using reporter dyes that are in proximity to one another in canonical nucleosomes (49,50). These changes are reversed when FACT dissociates from the complexes, although the extent to which H2A-H2B dimers are restored varies with the solution conditions (47). Mammalian Spt16:SSRP1 does not form stable complexes with nucleosomes unless a double-strand break is introduced or the DNA is truncated to expose the entry/exit point histone surfaces (34,48). However, addition of Nhp6 overcomes this barrier, causing mammalian FACT to alter nucleosome structure in a manner similar to yeast FACT (56). Both yeast and mammalian FACT are therefore capable of achieving full reorganization *in vitro*. Optimal levels of reorganization in both cases required about 2 μM Nhp6, but yeast nuclei are estimated to contain nearly 30 μM Nhp6 (56), so this requirement is not physiologically unrealistic.

FACT therefore comprises multiple histone-binding domains that can interact with surfaces that are inaccessible in intact nucleosomes, and HMGB-family proteins influence the ability to achieve or remain in the reorganized form. The binding sites on histone surfaces are sequentially exposed during reorganization and sequentially buried during resolution to assemble a nucleosome (22,24,33,56,59–60). The multiple, flexibly connected domains of FACT allow it to destabilize the nucleosome, tether the components together while enhancing accessibility of the DNA, and guide the process of assembly.

FACT also appears to be able to alter higher order chromatin structures beyond the nucleosome. Atomic force experiments show that prolonged incubation of FACT with nucleosome arrays weakens the stability of higher order tetranucleosome modules, and this appears to influence transcriptional regulation *in vivo* (61).

## FACT AS A TRANSCRIPTION INITIATION AND ELONGATION FACTOR

The Spt16 subunit of FACT was initially implicated in transcription initiation by genetic studies (10,62–63), but its purification as a factor that increased the rate of transcription on a nucleosomal template by human RNA Pol II *in vitro* suggested an additional role in elongation (1,46; but also see 64). However, it is important to note that TFIIS, LEDGF, HDGF2 and RSC (with Nap1) also have activity in this elongation assay, suggesting that polymerase progression *in vitro* can be enhanced through multiple mechanisms (64–66). Other evidence supporting a role during ongoing elongation includes the co-localization of FACT with RNA Pol II across the length of transcription units (5,67–73), and the correlation between FACT occupancy and the induction or repression of transcription (5,67). Proteomic studies also identified associations between FACT and components of transcription complexes (4,6,74–76), although direct interactions have not yet been demonstrated, so these associations may be indirect, possibly bridged through simultaneous interactions with histones.

It was initially proposed that FACT enhances elongation by converting nucleosomes encountered by RNA Pol II to hexasomes, thereby helping to overcome these barriers to progression (17). This model predicts that FACT deficiency should cause a decrease in the rate of transcription elongation (the number of nucleotides added to a growing transcript per unit of time); this prediction has not yet been tested *in vivo* by an accurate method for measuring the elongation rate genome-wide, but published attempts to validate this feature of the model at specific loci have not yet detected the expected changes (5,77–78). As FACT is not essential for proliferation in all circumstances, it cannot be a unique, obligate factor required to permit transcription elongation through chromatin as originally supposed. Overcoming nucleosomal barriers during polymerase progression therefore may not be the primary function of FACT, which raises the question of what that function is, as discussed further below.

Several lines of evidence support a role for FACT during initiation of transcription. Either overexpression of subunits or deficiency of FACT activity can cause inappropriate derepression of a transposon-associated promoter in yeast (10,79), and FACT mutants show an increase in transcripts from many genes (68,73,80–83). Derepression of transcription in its absence indicates that FACT has a global role in maintaining chromatin in a form that blocks inappropriate transcription initiation, described further in a later section. In addition to this role in maintaining repression, FACT also has a positive role in activating transcription. Early studies revealed that a FACT defect can cause a cell cycle delay due to inefficient activation of cyclin gene expression (62), and later studies showed that FACT contributes to the activation of several other inducible genes by enhancing nucleosome eviction, including *PHO5* (84), *HO* (19, 85) and *GAL1-GAL10* (18) in yeast, and *OCT4* in mammalian cells (20). Notably, FACT acts prior to RNA Pol II recruitment at several of these promoters, and FACT depletion strongly reduces the formation of preinitiation complexes globally (86). FACT can therefore be important for either maintaining chromatin barriers or for overcoming them outside of transcription units and prior to the initiation or elongation phases of transcription.

## EVIDENCE THAT FACT STABILIZES NUCLEOSOMES DURING OR AFTER TRANSCRIPTION

FACT’s ability to destabilize nucleosomes attracted early focus, but more recent work has emphasized the importance of FACT in forming or maintaining nucleosomes. The reversibility of reorganization and the ability of FACT to promote nucleosome assembly *in vitro* implied a potential role in assembling nucleosomes (2,17,49–50), and the multiple contacts between FACT domains and histones or DNA suggested that it could tether components together to enhance assembly (34,87). Direct evidence for nucleosome stabilization was observed in a detailed kinetic analysis of RNA Pol II progressing through a reconstituted nucleosome in the presence of FACT (21). In this case, pauses were observed at several sites within the nucleosome and FACT reduced the duration of some of these delays, but the overall effect of FACT was not to evict H2A-H2B but rather to promote survival of full nucleosomes by reducing the displacement of dimers.

Genome-wide measurements of the rate of histone turnover in yeast chromatin also support a role for FACT in nucleosome stabilization *in vivo*. FACT is essential for viability in *Saccharomyces cerevisiae*, but conditional removal of FACT activity can be achieved by a temperature shift of cells carrying the *spt16-G132D (spt16–197)* allele (88) (notably, FACT degradation appears to be reduced when transcription is blocked pharmacologically (73), suggesting an interplay between transcription and FACT stability). Acute loss of FACT using this method resulted in an increase in the rate of histone turnover, with the effects proportional to the level of transcription (8). Loss of a factor primarily involved in destabilizing or evicting nucleosomes would be expected to cause a decrease in the rate of histone turnover, so this result suggests that FACT makes a greater global contribution to nucleosome stabilization than to destabilization in yeast, and that this stabilization is particularly important for the survival of nucleosomes in transcriptionally active regions.

Genome-wide mapping of post-translational histone modification patterns and histone variant occupancy show that acute loss of FACT leads to general scrambling of the pre-existing chromatin architecture. For example, the variant H2A.Z is usually localized to the +1 nucleosome of genes, and patterns of other histone modifications are characteristic for different regions within transcription units, but these patterns all become more diffuse after loss of FACT (9,89). This shows that FACT is at least partly responsible for preventing dispersal of the components of nucleosomes during transcription, and therefore for allowing the local patterns of histone variants and modifications to resist disruption. This appears to have functional consequences, as FACT is implicated in establishing the promoter-proximal pausing that is typically associated with the +1 nucleosome in higher eukaryotes (90). FACT has also been shown to have a role in stabilizing nucleosomes and promoting epigenetic transmission of a heterochromatic state in the yeast *Schizosaccharomyces pombe* through a similar suppression of histone turnover (12,91). Blocking dispersion of modified histones is likely to depend on FACT’s ability to tether the components of a nucleosome together while it is transiently disassembled.

High transcription frequencies are generally associated with high rates of histone turnover and decreased nucleosome occupancy, suggesting that transcription itself may cause ‘erosion’ of chromatin (18,92), and FACT appears to have roles in both preventing this and in restoring the chromatin integrity if damage occurs. Induction of the *GAL1* gene causes reduced nucleosome occupancy in the gene body, and repopulation of these nucleosomes after repression is severely impaired in the absence of normal levels of FACT (18,77). Repopulation defects were also observed during the repression phase of a set of pleiotropic drug resistance genes using mutations that only partially disrupt FACT functions (93). Notably, these repopulation defects and those observed at the *HO* locus affected promoter regions upstream of the transcription start sites (19,85) indicating that FACT can affect both nucleosome eviction and reassembly in the absence of high levels of transcription. FACT is therefore important for restoring nucleosome occupancy whether the loss results from transcription or other mechanisms.

Failure of FACT to stabilize or restore nucleosomes during and after transcription has widespread consequences. Many genes harbor cryptic internal promoter elements whose repression depends on the rapid, efficient restoration of chromatin after passage of RNA Pol II (5,94–97). FACT defects lead to activation of these cryptic promoters, consistent with reduced restoration of nucleosomes in the wake of active transcription (5,94–95,98,99). Cryptic promoter activation can have functional consequences on the expression of local genes (for example, 100–102), but is likely to be an aberrant defect in most circumstances. In contrast, the normal regulation other genes like *SER3* depends directly on the efficiency of chromatin restoration during transcription elongation. In this case, transcription of the non-coding RNA from the upstream *SRG1* gene alters the pattern of nucleosome deposition over the *SER3* promoter, maintaining it in a repressed state (103,104). Loss of coupling between transcription and nucleosome deposition therefore leads to decreased nucleosome occupancy over the *SER3* promoter and increased transcription of this gene, as observed when FACT or histones are mutated (103,105–107). These examples illustrate the importance of restoring chromatin integrity after it is disturbed by transcription to prevent inappropriate promoter usage, and the role of FACT in supporting the reconstruction of disrupted chromatin.

One strand of the DNA is typically favored as the template for transcription, but suppressing the use of the other strand (preventing ‘antisense transcription’) can depend on the local chromatin architecture (for example, see 108). Antisense transcript production is elevated in FACT mutants, with the aberrant transcripts often being associated with the 5′ ends of genes (73,82,109–110). This has been attributed to derepression of cryptic antisense promoters in these regions (73) or to a reduced ability of the +1 nucleosome to terminate these transcripts (82). In any case, decreased FACT function reduces the stringency of control over transcription from both strands, leading to increased production of aberrant transcripts from many sources.

FACT binds to histone surfaces exposed by disruption of the canonical nucleosome structure *in vitro*, predicting that FACT localization might be driven by processes that disturb nucleosomes *in vivo*. This idea was tested by crosslinking FACT with chromatin and examining the nucleosomal DNA fragments associated with it after treating with MNase (71). The results showed preferential binding of FACT to non-canonical nucleosomal structures based on a different pattern of MNase sensitivity than found in bulk chromatin. Importantly, the changes in FACT occupancy observed after blocking transcription suggested that disruption of chromatin by elongating RNA Pol II is the primary driver of FACT localization, and therefore that FACT binds to chromatin after transcription disrupts the nucleosomes. This conclusion inverts the initial model, suggesting that instead of FACT being localized to transcription units to support RNA Pol II progression, it localizes to regions where histone surfaces that contain its binding sites have been exposed by disruption of nucleosomes during transcription. In this view, FACT is not recruited by interactions with the transcription machinery but by the effects of the passage of RNA polymerase on nucleosomal integrity. This does not rule out a role for FACT in initiation or elongation, but focuses attention on its functions in stabilizing nucleosomes or repairing chromatin that has been damaged by other processes (111) and raises the possibility that these could be its primary role in some circumstances.

## FACT AS A DNA REPLICATION FACTOR

In addition to roles in transcription, FACT was also identified as a DNA polymerase α binding protein that directly contacts the catalytic subunit Pol1 (Pob3 = polymerase one binding factor 3; Spt16 was Pob2 in this screen and the lagging-strand organizing factor Ctf4, with which FACT competes for binding to Pol1, was called Pob1; 112–114). FACT has also been reported to interact directly with the replicative helicase subunits MCM2 and MCM4 (3,115), and this interaction has been implicated in activating the helicase to promote progression of the replication fork (116). The interaction of FACT with MCM proteins may also contribute to the observed segregation bias of parental nucleosomes to the leading and lagging strands during replication (117). The ability of FACT to tether nucleosomal components together may therefore affect the survival of parental nucleosomes during replication as it does during transcription, with potential consequences for the stability of epigenetic states (12,83). The association with lagging strand factors like Pol1 and Ctf4 could reflect a role in *de novo* nucleosome assembly. Consistent with this, addition of purified FACT promoted rapid assembly of nucleosomes after DNA synthesis in a reconstituted replication complex *in vitro* (118).

Nascent chromatin is marked by acetylation of H3-K56, and FACT has been shown to have genetic interactions with the H3-K56 acetylation machinery, although unlike the Pob3 homolog Rtt106, FACT does not appear to directly read this mark (82,119–120). Many histone chaperones have been associated with chromatin deposition (121,122), and it remains unclear which roles might be unique and which are redundant. However, the *spt16-m* allele affects the region where FACT binding clashes with an H3-H4:DNA contact (48), and this mutation alters the deposition of nucleosomes coupled with replication (120). *spt16-m* also interacts genetically with other histone chaperones and alters the stability of complexes containing Rtt106 that are dependent on H3-K56 acetylation (120). Nascent chromatin also contains ubiquitylated H2B, and FACT collaborates *in vitro* and *in vivo* with Upb10, one of the proteases that can remove the H2B-K123Ub modification during the maturation of chromatin (123, and also see 110). These observations link FACT activity to DNA replication, possibly through a role in directly promoting progression of the replication fork through association with the MCM2–7 complex, but also by collaborating with a network of other histone chaperones and replication factors to enhance the assembly and maturation of chromatin with appropriate post-translational modifications in the wake of the fork.

## A UNIFYING MODEL FOR FACT FUNCTIONS

The multiple independent contacts between FACT domains and histone/DNA surfaces suggest that FACT competes with DNA to trap exposed histone surfaces, using a stepwise, ratcheting mechanism to disrupt existing nucleosomes and the reversal of these steps to assemble them (35; see Figures 1 and 2). The ability to disrupt nucleosomes could obviously contribute to deconstructing existing chromatin structures, but this model also provides the opportunity for FACT to monitor the structural integrity of nucleosomes during the resolution of the reorganized form, providing a check on chromatin quality. Yeast cells contain about 42 000 copies of FACT (87), enough to associate simultaneously with over half of the ∼70 000 nucleosomes in these cells (124,125). This level of abundance suggests that FACT can act globally in yeast cells, and we propose that one of its functions is to continuously monitor chromatin integrity genome-wide. The relative abundance of FACT in mammalian cells appears to be lower, although less quantitative information is available. HeLa cells were estimated to contain about 100 000 FACT molecules based on purification yield (46), which we calculate would be less than 1% of the number of nucleosomes. This difference in ratio could reflect the relative need for monitoring in the absence of widespread heterochromatin formation in yeast, the absence of some forms of chromatin modification, or other variables in forming stable chromatin or tolerating aberrations, as discussed below.

Specific mutations in FACT subunits cause different activity defects *in vitro*, and different phenotypes *in vivo*, indicating distinct activities and functional roles for individual domains (119,120). For example, FACT containing the mutant Pob3-Q308K protein binds to and reorganizes nucleosomes normally, but it fails to dissociate efficiently, leading to abnormal persistence of complexes (126). Notably, the homologous residue in human SSRP1 is in a loop that contacts DNA (45), and while both *pob3-Q308K* and *pob3-Q308R* were isolated multiple times in a screen for *pob3* mutations that cause sensitivity to the DNA replication toxin hydroxyurea, *pob3-Q308A* had no phenotype (127), suggesting that introduction of a positive charge near the DNA causes the reduced dissociation efficiency. In contrast, FACT with the Spt16–11 mutant subunit binds to and releases from nucleosomes normally but reorganizes them inefficiently (51). The phenotypes caused by these mutations are suppressed and enhanced by different profiles of histone mutations that affect different domains of nucleosomes (51,126). These and other genetic and biochemical studies suggest that promoting and resolving changes in nucleosome structure have separable functions in distinct processes *in vivo*. For example, reorganization of nucleosomes by FACT is expected to assist nucleosome eviction, and the resolution of reorganized complexes is expected to promote nucleosome deposition or stabilization.

Repeated cycles of reorganization and resolution could be used to monitor the integrity of the nucleosome by asking if its components are functional. In this view, the *pob3-Q308K* allele encodes a protein that has difficulty releasing from normal nucleosomes, triggering the signal that the nucleosome is abnormal and therefore blocking dissociation. Similarly, Pob3-Nhp6 fusion proteins reduced the displacement of H2A-H2B dimers during reorganization *in vitro*, which was interpreted as being a result of more efficient DNA bending in the final stage of nucleosome assembly allowing FACT to dissociate more quickly, spending less time in the reorganized state that is prone to dimer loss (56). In the model proposed, dissociation of FACT from a nucleosome requires its binding sites to be concealed, which happens only if a canonical structure with fully wrapped DNA is completed. Reversible reorganization followed by successful dissociation of FACT therefore provides a quality control step or ‘integrity checkpoint,’ as release signals the assembly of an intact nucleosome with appropriate properties. Recurring cycles of binding, reorganization, resolution, and release would then provide a mechanism for monitoring chromatin integrity.

Given FACT’s abundance, what is the role of its direct interactions with other proteins? The standard view is that interactions with DNA-binding protein transcription factors ‘recruit’ proteins like FACT to specific genomic sites. In a biophysical sense, recruitment means that when a protein diffuses to a region containing an anchoring interaction, it persists in the vicinity longer than it would in the absence of the interaction. However, the abundance of FACT suggests that its local concentration is always high throughout the nucleus in yeast cells, making increased persistence time a less effective way to regulate its functions. An alternative is that these interactions are instructive, changing the properties of FACT in local environments. For example, yeast FACT binds Swi6, and this interaction appears to contribute to a specific set of nucleosome evictions during the activation of the *HO* promoter (128). Perhaps Swi6-binding promotes FACT’s reorganization activity or suppresses its reassembly activity locally, increasing the probability of nucleosome eviction nearby. Association with Mcm2 might also promote nucleosome eviction during DNA replication, whereas binding to Pol1 could favor assembly. During eviction, FACT could tether the components of a specific nucleosome together to promote subsequent reassembly without dispersing modified histones, while during deposition it could cooperate with other histone chaperones by gathering histone subunits into sets with the correct stoichiometric ratios, or by providing a quality control monitor, with its tendency to provide a particular function influenced by its interactions with other proteins.

Overall, we propose that FACT can either initiate disruption of a nucleosome or promote the assembly of one, that the tendency to do one or the other can be influenced by local conditions or interaction with other factors, and that release of FACT from a nucleosome serves as a monitor for its integrity.

## WHY IS FACT ESSENTIAL IN SOME CELLS BUT NOT OTHERS?

Initial studies indicated that FACT is essential for viability in a range of eukaryotes, including vegetative growth of the yeast *S. cerevisiae*, and embryonic development of several plant and animal species (23,24). Broad roles in both transcription and replication provided multiple plausible explanations for this, as FACT appeared to have core roles in these essential processes. It was therefore surprising that deleting the gene encoding the Pob3 subunit of FACT in *S. pombe* caused a significant growth defect but was not lethal, whereas deletion of Spt16 could not be tolerated (129). Subsequent surveys of differentiated mammalian cell lines and tissues revealed that FACT abundance is variable, with little or no detectable FACT in many normal differentiated cell types, but high levels in many cancer cell lines and tumors (30,130). Deletion of the gene encoding Spt16 in mouse embryonic fibroblasts (MEFs) resulted in cells that grew at a normal rate but were unable to be induced to form pluripotent stem cells (iPSCs; 25). A class of small molecule drugs called curaxins trap FACT in complexes with chromatin, providing a pharmacological tool for disrupting FACT function (24,31). Curaxins show greater toxicity with cancer cell lines than with normal cells (24,31), and also block the transition of MEFs to iPSCs (25). These results indicate that both the level of FACT and the need for its functions vary with cell type, and that at least some types of differentiated cells appear to proliferate normally without FACT at all. FACT therefore cannot be a unique and essential core component of either the transcription or replication machinery, as previously supposed, forcing a significant reorientation of models for FACT function. What, then, are the core functions of FACT and why is the requirement for these functions variable among proliferating cells?

One potential explanation is that FACT’s function is always essential but can be supplied by other factors in some cells. LEDGF and HDGF2 were purified from cells lacking FACT using the original RNA Pol II elongation assay under this assumption (130), but it remains to be determined whether they display the synthetic lethality that would be expected if these represent factors that are redundant with FACT for an essential function. In addition, there may be cell-type specific redundancy between FACT and other histone chaperones. Another explanation would be that FACT is unique but its functions are only essential in a subset of circumstances. For example, FACT might be needed only when cells experience high levels of overall transcription, a high density of output from a subset of genes, or frequent cycles of replication. In these scenarios, transcription and replication can be accomplished without FACT if the rate at which they must be initiated or completed is below some threshold but not if the required output is higher.

Based on its activities *in vitro*, it seems likely that FACT’s essential function, in the cases where it is essential, is related to its ability to alter nucleosomal structures. If so, the question becomes, ‘how does FACT affect the integrity of chromatin and what sets the threshold for tolerating aberrations in this function?’ One possibility is that FACT is needed during development because its role in evicting nucleosomes is essential to navigate the transitions between transcription profiles that are needed as cells adopt different fates in an ordered sequence. This is consistent with the relocalization of FACT during heat shock in yeast as a new expression profile is adopted (131), but does not explain why FACT is essential for the viability of these cells or cancer cells under standard growth conditions. Another possibility is that cells have variable ability to tolerate the flaws in chromatin structure that occur when FACT is not available. By analogy to DNA repair mechanisms whose importance varies depending on the level of DNA damage, FACT could be part of a ‘chromatin repair’ pathway that monitors and maintains the integrity of the packaging of DNA rather than the DNA itself.

The need for a repair function would depend on the number of challenges to chromatin stability the cell faces and how well it can tolerate deviations such as nucleosome displacement from transcribed regions or the excess free histones that can accumulate in the absence of FACT activity (132). The high density of transcription units in yeast genomes makes it more likely that a gene has a nearby neighbor, which might make mechanisms for limiting the effects of transcription on those neighbors more important than it is in more sparsely populated genomes. It is notable in this context that FACT localization and effects are not limited to genes transcribed by RNA Pol II, but also include the heavily transcribed (and in the case of rDNA, tandemly repeated) targets of RNA Pol I and RNA Pol III as well (133). Both yeasts and cancer cells undergo rapid cycles of replication that stress the ability to assemble mature chromatin, and chromatin in these cells could be inherently fragile due to decreased stability of nucleosomes formed with yeast histones, or the mutations in histone genes associated with many cancer cells (134). Chromatin in these cells might therefore be more frequently or more easily damaged, leading to greater dependence on repair mechanisms. Alternatively, these cells might be inherently less able to tolerate the consequences of decreased chromatin integrity, such as elevated levels of cryptic promoter activation, increased expression of genes that should be repressed, or the inability to activate repressed genes in response to environmental cues. These flaws might be tolerable under steady-state growth conditions in an optimal environment, but could be cumulatively less tolerable if nutrients are scarce, the conditions warrant an adjustment in the gene expression profile, or if rapid proliferation is required.

Cancer cells often have reduced DNA damage repair checkpoint activity, including frequent loss of components of the p53 pathway (135). This illustrates the principle that cells are generally more careful than is absolutely necessary, creating a potential selective advantage for more reckless behavior. The increased mutation frequencies and decreased overall genomic stability associated with loss of checkpoints allow cancer cells to evolve rapidly to overcome barriers that normally restrain inappropriate proliferation, but it also makes them less able to tolerate additional DNA damage and therefore more dependent on DNA repair (136,137). Similarly, less stringent control of gene expression might be suboptimal, but tolerable to different extents in different types of cells. DNA damage response checkpoints have been studied mainly as sensors of the integrity of DNA itself, but formation of mature chromatin has been observed to be part of the signal for turning off these alarms (138). This suggests that checkpoints might be needed to monitor the integrity of chromatin, making FACT and other factors that prevent chromatin damage or repair it more important in cells lacking these control mechanisms.

The integrity of higher-order chromatin structures is presumably at least partially dependent on the quality of nucleosomes. These structures contribute to the regulation of origin firing and the accounting process that limits DNA replication to ‘once, and only once’ per S phase, and to the compaction and segregation of chromosomes during mitosis (139). Cancer cells also tend to lack S phase progression and G2/M monitoring checkpoints, suggesting they would also be more sensitive than normal to decreased chromatin quality. Consistent with this, cancer cells often have aberrant patterns of histone modifications and altered higher order chromatin architecture, and the curaxins that block FACT function can disrupt this architecture more readily than they do in normal cells (29). FACT also appears to contribute to chromosome architecture more directly by influencing cohesin function (140). Cells with other challenges to chromatin compaction or a greater need to manage higher-order structural organization of the genome within the nucleus might therefore have greater need for FACT to provide for adequate regulation of cohesin functions.

Variable need for FACT among mammalian cell types could then result from a threshold for tolerating an increase in the level of chromatin disruption that occurs in the absence of FACT combined with the impaired ability to repair this damage. ‘Chromatin quality’ comprises many layers of components, and different organisms have different numbers of layers. For example, budding yeast lack DNA methylation and certain histone modification systems found in mammalian cells that could provide additional stability to chromatin structure, potentially making chromatin repair more necessary in this species of yeast.

## SUMMARY

FACT is a histone chaperone capable of managing the equilibrium between intact nucleosomes and their components. This activity makes it useful for evicting nucleosomes or assembling them, and these activities are likely to be useful in distinct processes such as activating transcription or repressing it. The abundance and requirement for FACT varies among cell types, and may depend on the ability of each type of cell to tolerate disrupted chromatin architecture or chromatin damage, making cells more or less reliant on FACT to prevent these disruptions from occurring or to contribute to repairing them. The proposed role of FACT in chromatin repair might therefore provide a central framework for understanding its broad range of effects in diverse processes.
